# Prospective Comparison of the Performance of MRI Versus CT in the Detection and Evaluation of Peritoneal Surface Malignancies

**DOI:** 10.3390/cancers14133179

**Published:** 2022-06-29

**Authors:** Claramae Shulyn Chia, Louis Choon Kit Wong, Tiffany Priyanthi Hennedige, Whee Sze Ong, Hong-Yuan Zhu, Grace Hwei Ching Tan, Jin Wei Kwek, Chin Jin Seo, Jolene Si Min Wong, Chin-Ann Johnny Ong, Choon Hua Thng, Khee Chee Soo, Melissa Ching Ching Teo

**Affiliations:** 1 Department of Sarcoma, Peritoneal and Rare Tumours (SPRinT), Division of Surgery and Surgical Oncology, National Cancer Centre Singapore, Singapore 169610, Singapore; louis.wong14@sps.nus.edu.sg (L.C.K.W.); zhu.hong.yuan@nccs.com.sg (H.-Y.Z.); grace.tan.h.c@singhealth.com.sg (G.H.C.T.); seo.chin.jin@singhealth.com.sg (C.J.S.); jolene.wong.s.m@singhealth.com.sg (J.S.M.W.); johnny.ong.c.a@singhealth.com.sg (C.-A.J.O.); soo.khee.chee@singhealth.com.sg (K.C.S.); melissa.teo.c.c@singhealth.com.sg (M.C.C.T.); 2 Department of Sarcoma, Peritoneal and Rare Tumours (SPRinT), Division of Surgery and Surgical Oncology, Singapore General Hospital, Singapore 169608, Singapore; 3 SingHealth Duke-NUS Oncology Academic Clinical Program, Duke-NUS Medical School, Singapore 169857, Singapore; 4 SingHealth Duke-NUS Surgery Academic Clinical Program, Duke-NUS Medical School, Singapore 169857, Singapore; 5 Duke-NUS Medical School, Singapore 169857, Singapore; 6 Division of Oncological Imaging, National Cancer Centre Singapore, Singapore 169610, Singapore; hennedige.tiffany.priyanthi@singhealth.com.sg (T.P.H.); kwek.jin.wei@singhealth.com.sg (J.W.K.); thng.choon.hua@singhealth.com.sg (C.H.T.); 7 Division of Clinical Trials and Epidemiological Sciences, National Cancer Centre Singapore, Singapore 169610, Singapore; ong.whee.sze@singhealth.com.sg; 8 Laboratory of Applied Human Genetics, Division of Medical Sciences, National Cancer Centre Singapore, Singapore 169610, Singapore; 9 Institute of Molecular and Cell Biology, A*STAR Research Entities, Singapore 138673, Singapore

**Keywords:** computed tomography, magnetic resonance imaging, peritoneal metastases, peritoneal cancer index, cytoreductive surgery, intraperitoneal chemotherapy

## Abstract

**Simple Summary:**

Early diagnosis, evaluation, and appropriate treatment of peritoneal surface malignancies remains difficult. While computed tomography and magnetic resonance imaging are commonly used for these purposes, it remains unknown whether one is superior to the other. The aim of the current study was therefore to prospectively compare the two imaging modalities, using intra-operative evaluation as a reference standard. Our findings indicate that they are comparable in the detection and evaluation of peritoneal surface malignancies. Thus, either imaging modality may be appropriate, depending on clinical indications and resource management. It should however be noted that both modalities are likely to underestimate the true burden of disease, which may negatively impact treatment decisions.

**Abstract:**

Background: The performance of MRI versus CT in the detection and evaluation of peritoneal surface malignancies (PSM) remains unclear in the current literature. Our study is the first prospective study in an Asian center comparing the two imaging modalities, validated against intra-operative findings. Methods: A total of 36 patients with PSM eligible for CRS-HIPEC underwent both MRI and CT scans up to 6 weeks before the operation. The scans were assessed for the presence and distribution of PSM and scored using the peritoneal cancer index (PCI), which were compared against PCI determined at surgery. Results: Both MRI and CT were 100% sensitive and specific in detecting the overall presence of PSM. Across all peritoneal regions, the sensitivity and specificity for PSM detection was 49.1% and 93.0% for MRI, compared to 47.8% and 95.1% for CT (*p* = 0.76). MRI was more sensitive than CT for small bowel disease, although the difference did not reach statistical significance. Comparing PCI on imaging with intra-operative PCI, the mean difference was found to be −3.4 ± 5.4 (*p* < 0.01) for MRI, and −3.9 ± 4.1 (*p* < 0.01) for CT. The correlation between imaging and intra-operative PCI was poor, with a concordance coefficient of 0.76 and 0.79 for MRI and CT, respectively. Within individual peritoneal regions, there was also poor agreement between imaging and intra-operative PCI for both modalities, other than in regions 1 and 3. Conclusion: MRI and CT are comparable in the detection and evaluation of PSM. While sensitive in the overall detection of PSM, they are likely to underestimate the true disease burden.

## 1. Introduction

Cytoreductive surgery and hyperthermic intraperitoneal chemotherapy (CRS-HIPEC) play an important role in the treatment of various peritoneal surface malignancies (PSM), such as pseudomyxoma peritonei, peritoneal mesothelioma, and peritoneal metastases from colorectal cancer [1,2]. Its use is currently being evaluated in PSM arising from ovarian, gastric, as well as other less common primaries [3,4,5]. Factors predictive of improved overall and disease-free survival in patients undergoing CRS-HIPEC include the primary tumor histology, the extent of peritoneal disease, as well as the completeness of cytoreduction [6].

The extent of peritoneal disease is most commonly measured using the peritoneal cancer index (PCI). It ranges from 0 to 39 and is calculated by evaluating the distribution and size of peritoneal deposits in 13 abdominal regions [7]. The burden of disease as measured by the PCI is critical in clinical decision-making and surgical planning, as a high PCI or extensive small bowel involvement may prohibit complete cytoreduction. However, a major limitation is that the PCI can usually only be accurately determined intra-operatively, and any underestimation may lead to abandonment of the procedure and a change in treatment course to a palliative one instead. There is thus a pressing need to better identify which PSM patients are suitable candidates for curative resection on pre-operative assessment, so as to reduce unnecessary procedures and psychological stress for those patients, as well as to enable better utilization of operating theatre resources.

There has been attempts to characterize disease burden pre-operatively using various radiological imaging modalities. While the field has seen significant technological advances over the past decade, the achievable accuracy in mapping PSM with currently available methods remains uncertain. Technical challenges arise in part due to a complex peritoneal anatomy, as well as the extensive surface area capable of harboring small tumor deposits [8]. In practice, computed tomography (CT) scans are commonly used for this purpose, given their speed and high spatial resolution, as is the case in most other intra-abdominal malignancies. However, CT is limited by poor soft tissue contrast and therefore may be less sensitive in the detection of smaller nodules; prior studies by Coakley et al. (2002) and Low et al. (1997) showed that the sensitivity of CT for identifying tumors less than 1 cm was only 22% to 50% [9,10]. On the other hand, magnetic resonance imaging (MRI) scans provide superior soft tissue contrast and may be more effective in this aspect [11,12]. Previous reports are however conflicting; while some studies have shown that the sensitivity of MRI is higher than that of CT scans for picking up peritoneal disease [13,14,15], others found their performance comparable [16,17,18].

Furthermore, the interpretation of many previous studies investigating the two imaging modalities can be difficult due to a number of issues, including a lack of direct comparison, rapidly evolving imaging technology and protocols, variations in the protocols used, as well as the lack of or imprecise PCI quantification. Additionally, evidence in Asian populations is scarce. Thus, the current study aimed to compare the performance of MRI versus CT in the detection and evaluation of PSM, using a prospective design with predefined modern imaging protocols and PCI determined at surgical exploration as a reference standard.

## 2. Methods

### 2.1. Study Design

This is a prospective comparative diagnostic accuracy study with ethical approval by the SingHealth Institutional Review Board. Informed consent was obtained for all included participants. Patients with PSM undergoing CRS-HIPEC at the National Cancer Centre Singapore (NCCS) were recruited from 2016 to 2017, after assessment for suitability of treatment by a multidisciplinary tumor board. Additional eligibility criteria included fitness for surgery and no contraindications to CT or MRI scans. Those with extensive disease deemed inoperable on imaging were excluded. Included patients underwent both CT as well as MRI in the 6 weeks prior to surgery, to ensure disease evaluation at the same time point and minimal delay between imaging and surgery to avoid interim disease progression.

### 2.2. Imaging Protocol

CT scans were performed using either a GE Lightspeed 64 slice CT system or GE Resolution GSI Dual Energy 128 slice CT system. Patients were fasted 4 hours prior to the scan time, then given 800 mL of oral diluted iodinated contrast with 100 mL of IV iodinated contrast at a rate of 1 mL/sec prior to scanning. Images were acquired in a helical scan mode at 120 kV and 200 mA, with a slice thickness of 2.5 mm.

MRI scans were performed using a Siemens Magnetom Aera 1.5T (Siemens Healthcare, Erlangen, Germany). Intravenous Gadoterate Meglumine (Dotarem) was administered at 0.2 mL/kg. The following precontrast sequences were obtained: Axial HASTE 5-mm (abdomen), coronal HASTE 3-mm (abdomen/pelvis), axial VIBE 3-mm in-opp (abdomen), axial VIBE 3-mm (abdomen), axial DWI b50, 600 5-mm (abdomen), axial T1/T2 5-mm (pelvis), axial DWI b50, 800 5-mm (pelvis), and axial T1 VIBE 3-mm (pelvis). Following contrast administration, axial VIBE 3-mm (dynamic 3 phase-abdomen), coronal VIBE 3-mm (abdomen/pelvis), axial VIBE 3-mm (pelvis), and axial T1 delayed 3-mm (abdomen) sequences were obtained. The patient was then put in a prone position, and the following sequences were repeated: axial T1 VIBE 3-mm (abdomen) and axial T1 VIBE (pelvis).

### 2.3. Image Analysis

All CT and MRI images were independently read by one of three radiologists experienced in abdominal imaging, who were blinded to patient characteristics as well as surgical findings. Discrepancies between reads were reviewed and settled by consensus. Findings were documented in a standardized template. The PCI was scored radiologically by individually evaluating each of 13 abdominal regions for the presence and size of tumor implants: 0 for no visible implants, 1 for implants less than 0.5 cm, 2 for implants 0.5 cm to 5 cm, and 3 for implants greater than 5 cm or with the presence of confluent implants.

### 2.4. Surgical Exploration

During surgery, a midline laparotomy was used for abdominal access. All adhesions were lysed and the abdominal cavity was thoroughly evaluated for extent and sites of disease involvement. The PCI determined at surgical exploration formed the reference standard that the PCI determined on imaging was compared against. CRS was subsequently performed using standardized technique as described by Sugarbaker [19]. HIPEC was administered for 60 min at 42 °C, with either cisplatin or mitomycin-c, depending on primary tumor histology.

### 2.5. Statistical Analysis

The predicted sensitivity of MRI and CT in PSM detection was taken to be 95% and 55%, respectively, based on previous studies [13]. Historically, about 86% of our patients undergoing exploratory laparotomy underwent CRS-HIPEC. In order to detect a 40% difference in sensitivity at 80% power and 0.05 alpha using a two-sided McNemar’s test, at least 30 patients were needed after accounting for dropouts.

McNemar’s test was used to compare the sensitivity between MRI and CT in PSM detection while the paired t-test was used to compare differences between imaging and intra-operative PCI. The extent of agreement between the imaging and intra-operative PCI was evaluated based on the concordance correlation coefficient, with good agreement defined as a coefficient greater than 0.95. Within each PCI region, the weighted Cohen’s kappa statistic was used to assess the extent of agreement between the imaging and intra-operative PCI, with good agreement defined as a kappa of greater than 0.6. A 2-sided *p*-value of less than 0.05 was considered statistically significant. All analyses were performed using SAS version 9.4 (SAS Institute Inc., Cary, NC, USA).

## 3. Results

### 3.1. Baseline Characteristics of Study Cohort

**Figure 1:**
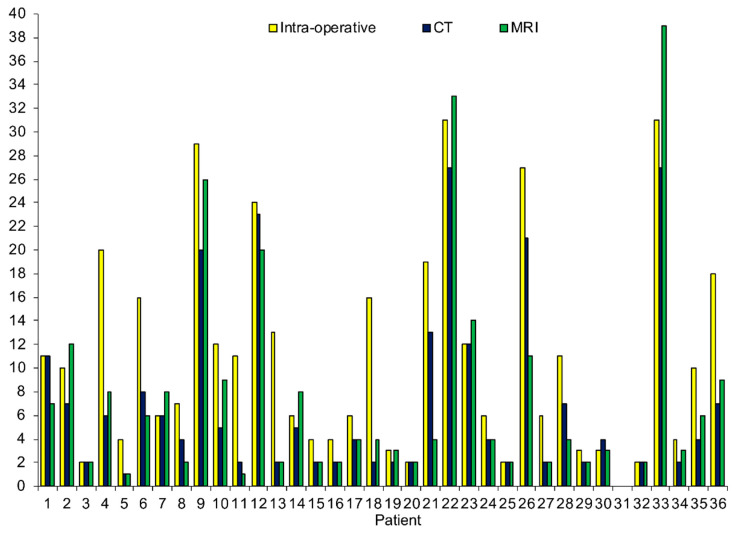
Total peritoneal cancer index score by individual patient.

All 36 patients with PSM recruited for this study were included in the final cohort and underwent pre-operative imaging with MRI and CT. There were 12 males and 24 females, with a mean age of 56.7 ± 10.9 years. Primary tumor origin included colorectal (44.4%), appendiceal (22.2%), ovarian (16.7%), as well as others (16.7%). A total of 75.0% of patients had prior surgery and 63.9% received neoadjuvant chemotherapy before CRS-HIPEC. 86.1% of all patients subsequently underwent successful CRS-HIPEC, with 87.5% achieving CC-0 cytoreduction. The PCI as determined by MRI, CT, and intra-operative exploration for individual patients is shown in Figure 1.

### 3.2. PSM Detection

Both MRI and CT were 100% sensitive and specific in establishing the diagnosis of PSM in individual patients. When averaged across all peritoneal regions, there were no significant differences between the two imaging modalities in the detection of PSM, with an overall sensitivity and specificity of 49.1% and 93.0% for MRI, compared to 47.8% and 95.1% for CT (*p* = 0.76; Table 1). Within each of the individual 13 peritoneal regions, there were also no significant differences in sensitivity between MRI and CT. However, it was found that imaging performance varied between specific peritoneal regions for both imaging modalities, with a higher sensitivity for tumor detection that ranged between 58% to 79% in PCI region 1 (right upper), region 3 (left upper), region 4 (left flank), region 5 (left lower), region 6 (pelvis), region 7 (right lower), as well as region 8 (right flank). Performance in the small bowel, corresponding to regions 9 to 12 (jejunum and ileum), was poor for both MRI and CT; while MRI was noted to be more sensitive (19% to 31%) compared to CT (0% to 7%) in these regions, the difference did not reach statistical significance.

### 3.3. Evaluation of PCI

**Figure 2:**
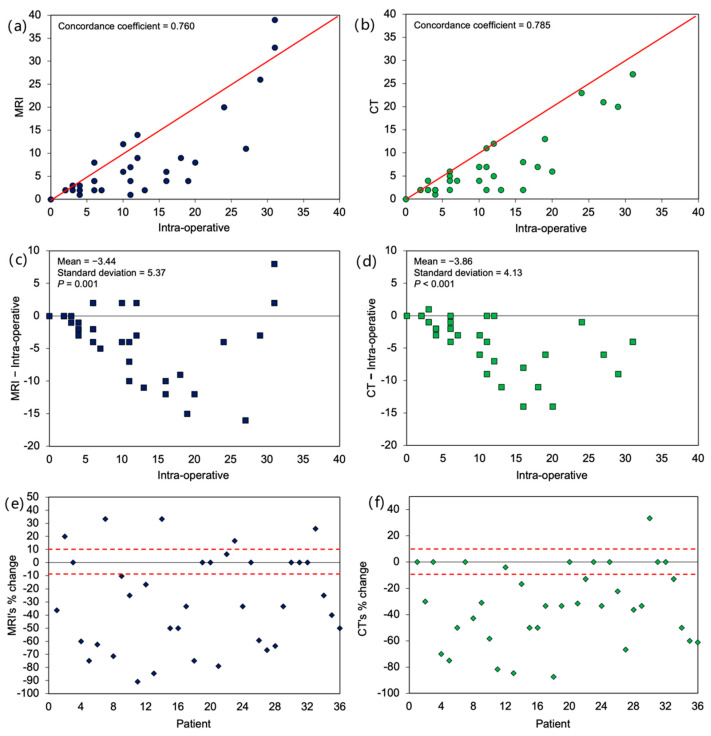
Agreement between imaging and intra-operative total peritoneal cancer index score. Scatterplot of (**a**) MRI and (**b**) CT against intra-operative score. Scatterplot of difference from intra-operative score for (**c**) MRI and (**d**) CT. Percentage change of (**e**) MRI and (**f**) CT score from intra-operative score by individual patients with 10% accuracy bound. *p*-values in (**b**,**c**) were based on paired *t*-test, testing whether mean differences were different from zero.

In terms of overall PCI assessment, it was found that the agreement between PCI determined on imaging versus intra-operative PCI was poor. The concordance coefficient was 0.76 and 0.79 for MRI and CT, respectively, with most cases deviating from the 45-degree agreement line (Figure 2a,b). Most often the imaging PCI was an underestimation of intra-operative PCI, with a mean difference of −3.4 ± 5.4 (*p* < 0.01) for MRI and −3.9 ± 4.1 (*p* < 0.01) for CT, respectively (Figure 2c,d). Additionally, there was an increase in absolute bias with higher intra-operative PCI (i.e., imaging scans were less accurate for patients with more extensive disease). The 10% accuracy bound for relative bias was also noted to be low: 22% for MRI and 25% for CT (Figure 2e,f).

Within individual peritoneal regions, there was similarly poor agreement between the imaging and intra-operative PCI for both MRI and CT (Table 2), where the weighted kappa statistics were less than 0.6 for all regions except region 1 (right upper) and region 3 (left upper). Between the two imaging modalities, the weighted kappa statistics were not significantly different from each other.

## 4. Discussion

Modern radiological methods continue to play an indispensable role in the diagnosis and management of various malignancies. Recent advances in imaging technology and techniques have enabled increasingly accurate visualization of diseases that were previously difficult to capture, of which PSM is one. In the pre-operative setting, accurate characterization of PSM is crucial to identify patients in whom CRS-HIPEC is a viable treatment option, given the fact that both the extent as well as the location of disease affects surgical resectability [20]. The optimal approach to evaluating such patients is an area of active research, although currently few proposed solutions exist. Diagnostic laparoscopy prior to laparotomy is one such option to evaluate the extent of disease. However, it is only feasible in a small subset of PSM patients, given that many would have had multiple previous surgeries with extensive intra-abdominal adhesions, resulting in difficult access. Additionally, it remains an invasive procedure requiring the induction of general anesthesia and operating theatre usage. The Peritoneal Surface Disease Severity Score (PSDSS) system, using a combination of clinical and histological parameters with imaging, has been proposed, although subsequent studies noted that 32% of patients classified as low disease burden using the system nevertheless underwent an “open and close” procedure [21,22]. Furthermore, assessment of the PCI using imaging methods is also an essential component of scoring the PSDSS.

To this end, radiological imaging remains the cornerstone of assessment for all PSM patients being evaluated for treatment. However, there is considerable debate regarding which imaging modality is best suited for this purpose. Overall, the current study did not identify a significant difference between MRI and CT in terms of overall PSM detection as well as in individual peritoneal regions, which is consistent with some previous studies but not others [13,16,17,23]. In terms of patient-based analyses, previous works reported a sensitivity and specificity between 43–100% and 40–100% for CT [9,24,25,26,27,28,29], compared to 84–90% and 82–95.5% for MRI [26,30,31]. In the meta-analysis by Laghi et al. (2017), it was found that CT had a pooled sensitivity of 83% and specificity of 86% in the diagnosis of PSM on a per-patient basis, compared to 86% and 88% for MRI [32]. For region-based analyses, sensitivities and specificities of between 24.5–96% and 72–98.1% were reported for CT [13,16,23,27,28,33,34,35,36,37], compared to 87–98% and 70–93.2% for MRI [13,16,23,38,39]. The pooled per-region sensitivity and specificity of CT was 68% and 88%, with 91% and 85% for MRI, as noted in a separate meta-analysis by Van’t Sant et al. (2020) [40].

Various studies have also reported decreased sensitivity for CT in the detection of disease in specific peritoneal regions, most commonly of the small bowel corresponding to regions 9 to 12, which is consistent with our observations. Choi et al. (2011) found that while overall sensitivity for PSM detection across all regions was 45%, sensitivity in the porta hepatis, lesser sac, small bowel mesentery, omentum, and bladder dome was less than 30% [34]. Similarly, Dresen et al. (2019) reported an overall sensitivity of 43.2% for CT; it was noticeably poorer for disease in the small bowel, in particular regions 9, 10, and 11, ranging from 17.7 to 33.3% [23]. In a separate study, it was found that 42.3% of all false-negative sites were bowel-related and 24.4% were mesentery-related [36]. Additionally, detection of tumors located on the small bowel and mesentery can also be hindered by significant intra-observer differences [27]. Several groups investigating the use of MRI have therefore suggested it may be more useful in this aspect. With the use of diffusion-weighted (DW) sequences on MRI, Michielsen et al. (2014) were able to achieve enhanced detection of small bowel and colonic mesenteric and serosal metastases when compared with CT, with a sensitivity of 50% versus 17% in small bowel serosa, 100% versus 67% in small bowel mesentery, 82% versus 45% in colonic serosa, and 83% versus 50% in colonic mesentery [15]. In a comparative study by Low et al. (2015), the performance of CT for small bowel was poor, particularly in regions 9, 10, and 11, with a sensitivity of 7%, 13%, and 7%. MRI however performed well, with corresponding values of 86%, 100%, and 100% [13].

Furthermore, it has also been noted in various studies that the sensitivity of both imaging modalities is strongly influenced by lesion size. In the study by de Bree et al. (2004), individual peritoneal implants were detected on CT at a rate of 9.1–24.3% for implants <1 cm, 14.3–28.2% for implants 1 to 5 cm, and 59.3–66.7% for implants size >5 cm [27]. Several other reports have used cut-offs between 0.5 and 1 cm: Metser et al. (2011) found that CT sensitivity decreased from 89.3% for implants ≥ 1 cm to 65.5% for implants <1 cm, while Marin et al. (2009) found that implants ≥ 0.5 cm had a sensitivity of 89% compared to 43% for implants <0.5 cm [35,36]. Similar results have been found for MRI, where lesions <1 cm had a sensitivity of 50%, in contrast with those ≥ 1 cm, where it was 93% [31]. Low et al. (2012) additionally identified a comparable linear relationship, where MRI sensitivity for implants < 0.5 cm was 78%, compared to 88% for implants 0.5 to 5 cm and 97% for implants >5 cm [38].

In terms of the assessment of disease volume, some studies have found that both CT and MRI tend to underestimate the true burden of disease to varying degrees. In the study by Dohan et al., CT-PCI underestimated the intra-operative PCI 75% of the time, with an absolute mean PCI difference of 4.89 [41]. A separate study noted that the mean CT-PCI was 8.7 when the mean intra-operative PCI was 12.9 [42]. Nevertheless, several other studies reported that there was good correlation between CT-PCI and intra-operative PCI, even when underestimation of the latter was present (r^2^ = 0.67–0.85) [37,43]. Other authors have suggested that MRI may perform better in this regard: Low et al. (2012), for example, found no statistically significant difference between MRI-PCI and intra-operative PCI [38]. The PCI scores were identical in 24% of the patients, had a difference of <5 in 49% of patients, and a difference of 5 to 10 in 27% of patients. When considering small, moderate and large volume disease, MRI was able to correctly predict the category in 88% of patients. A later study by the same group noted that the median percentage difference between the intra-operative PCI and the CT-PCI was 50%, compared with only 6% for the MRI-PCI [13]. In contrast to these studies, Mikkelsen et al. (2021) found that underestimation was present in both imaging modalities. They noted that the mean differences between the intra-operative PCI and the imaging PCI were 4.2 for CT and 4.4 for DW-MRI, with no statistically significant differences [17]. Additionally, the highest agreement between intra-operative and imaging PCI was seen when the burden of disease was low. These findings are in line with our observations. Possible reasons for the lack of difference between CT and MRI in the current study may be attributed to the use of updated CT scan protocols and techniques that facilitate more sensitive image capture, as well as the reading of scans by specialized abdominal imaging radiologists.

Overall, it can be seen that the choice between CT and MRI remains difficult with currently available evidence. In our experience, much of this stems from the fact that each comes with its own set of advantages and disadvantages. CT remains the preferred modality in most institutions, due to its wide availability and ease of access, rapid image acquisition time, as well as excellent spatial resolution. As previously mentioned, CT however suffers in performance with decreasing lesion size, as well as with location in certain sites, such as the mesentery, omenta, diaphragmatic surfaces, and small bowel serosa, in part due to its limited contrast resolution [27,44]. As a result, the underestimation of disease volume by CT has been well documented [13].

**Figure 3:**
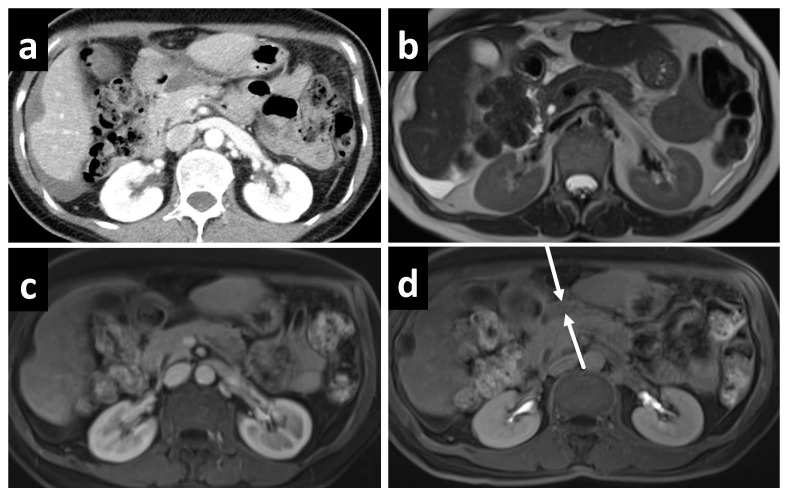
A 61-year-old female with mucinous appendiceal neoplasm, a small amount of fluid is noted within the lesser sac on (**a**) CT and (**b**) T2W MRI. Enhancement within the fluid, however, is barely appreciated on the (**c**) portal venous phase of the MRI, but becomes clearly apparent on the (**d**) delayed MRI.

**Figure 4:**
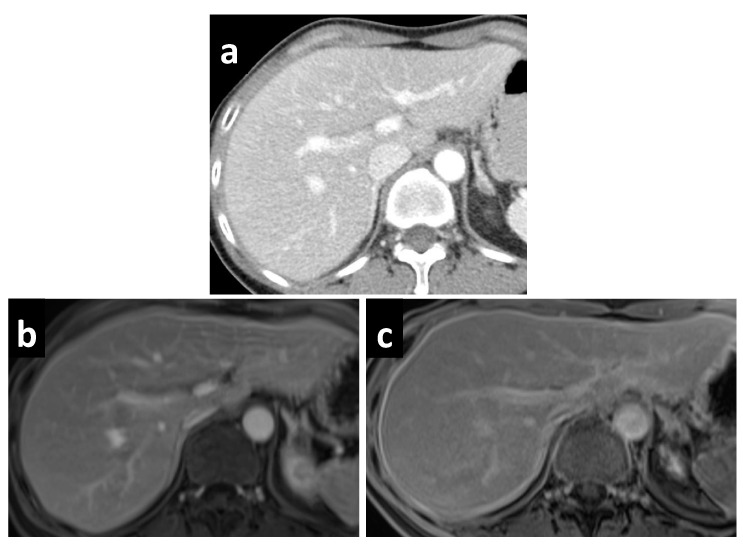
A 55-year-old female with PMP. (**a**) CT reveals no significant abnormality, but MRI performed on the same day demonstrates a thin enhancing line surrounding the liver on the (**b**) portal venous phase, which becomes more apparent on the (**c**) delayed phase.

**Figure 5:**
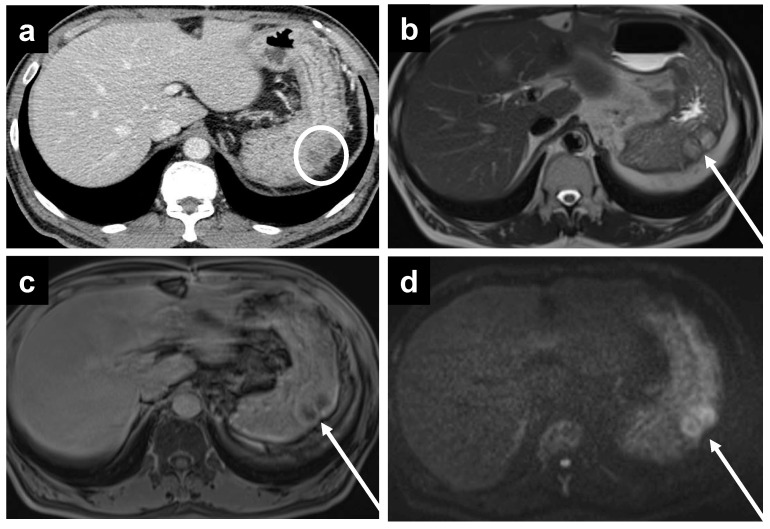
A 67-year-old male with metastatic colonic adenocarcinoma and gastric serosa peritoneal deposits. The deposits are not as well seen on (**a**) CT, as denoted by the white circle, but are more apparent on MRI, including the (**b**) T2W, (**c**) delayed, and (**d**) DWI sequences secondary to the superior soft tissue contrast.

**Figure 6:**
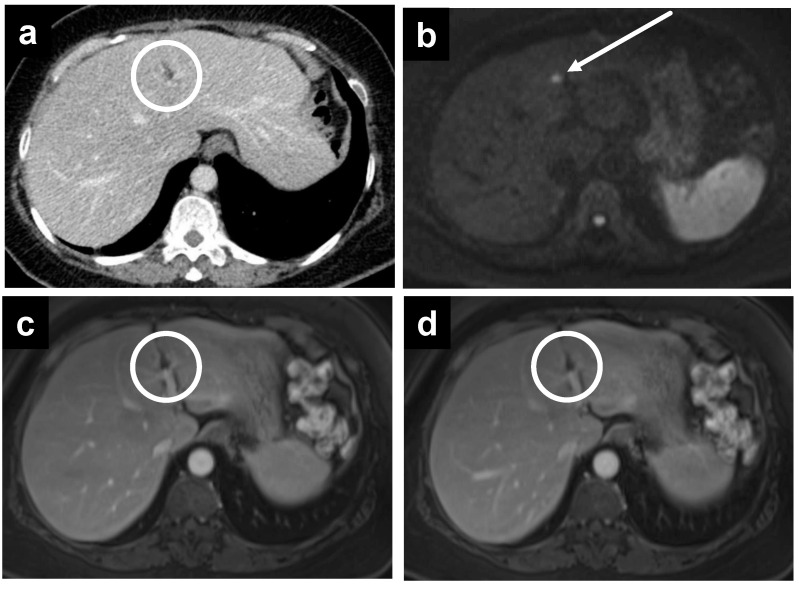
A 61-year-old female with subtle recurrent primary peritoneal carcinoma, a tiny deposit is only well seen on the (**b**) DWI as a hyperintense focus at the falciform ligament (white arrow). A rim-enhancing nodule is barely seen on (**a**) CT, as well as the (**c**) portal venous and (**d**) delayed phases on MRI (white circles).

**Figure 7:**
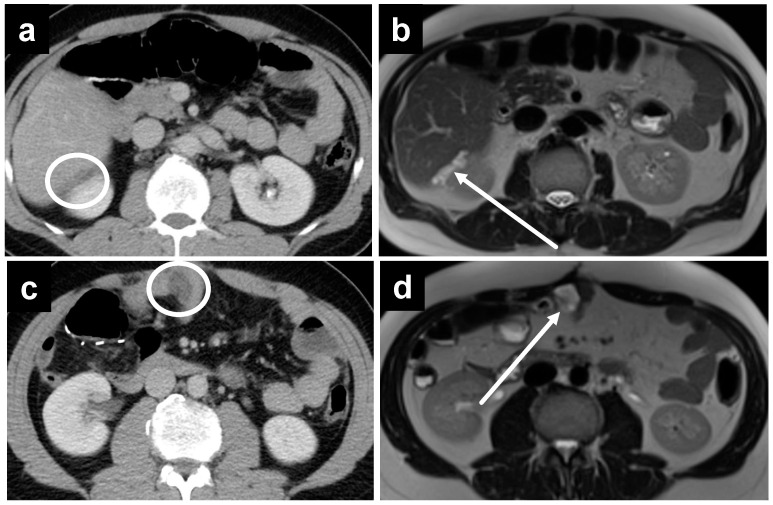
A 44-year-old male with recurrent mucinous appendiceal tumor; the hypodense fluid is not well seen on the (**a**) CT at Morrison’s pouch but easily seen on the (**b**) T2W MRI, where the fluid also appears to be loculated, suggestive of a tumor deposit as opposed to bland ascites. Similarly, the pocket of fluid among small bowel loops is not as well appreciated on (**c**) CT but is well seen on (**d**) T2W MRI.

On the other hand, the role of MRI in the evaluation of PSM has rapidly expanded over the past decade, in part due to greater access and streamlined protocols. The use of gadolinium contrast, fat suppression, and various imaging sequences has allowed for better visualization of the subtle changes in PSM. Most commonly in the evaluation of PSM, a combination of T1- and T2-weighted, delayed gadolinium-enhanced, and DW sequences are used. Peritoneal tumors exhibit optimal enhancement about 5 min following intravenous gadolinium injection, resulting in a high contrast conspicuity that makes delayed contrast-enhanced sequences excellent for detecting both small deposits as well as those in anatomically difficult sites, such as in the lesser sac (Figure 3). For perihepatic disease, enhancement greater than that of liver is a sensitive indicator, easily depicted on delayed contrast-enhanced MRI but not appreciated on contrast-enhanced CT (Figure 4.) The superior contrast resolution of MRI also allows for the detection of deposits located adjacent to soft tissue, such as the stomach (Figure 5). Additionally, most solid tumors exhibit restricted water diffusion due to their high cellularity and increased cell membrane per unit volume, which can be clearly picked up as high intensity lesions on DW images with excellent contrast resolution (Figure 6). Site-specific tumor deposits may therefore be better evaluated on DW images, in particular those within the mesentery, bowel serosa, perihepatic, and peripancreatic spaces [45]. For cystic/mucinous deposits, T2-weighted sequences are ideal; these lesions will appear as hyperintense foci that are often easily overlooked in CT images (Figure 7).

**Figure 8:**
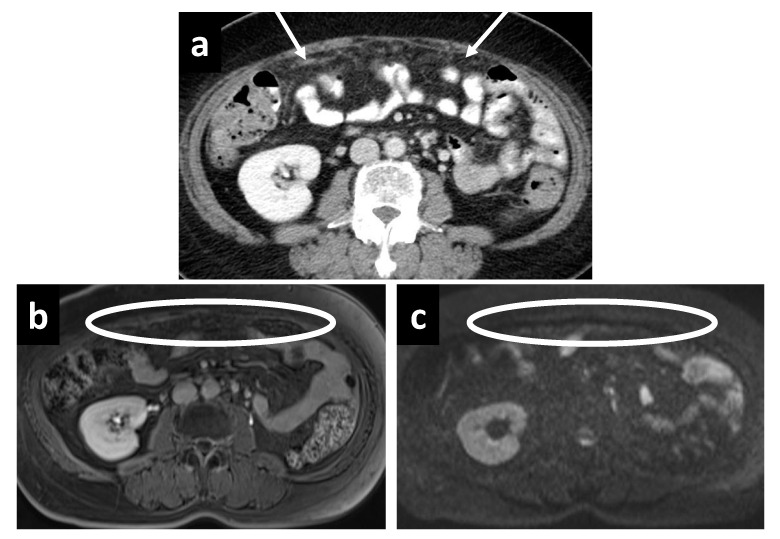
The superior spatial resolution of (**a**) CT is demonstrated here as irregular soft tissue stranding within the greater omentum, which is not appreciated on (**b**) delayed and (**c**) DW-MRI performed on the same day.

**Figure 9:**
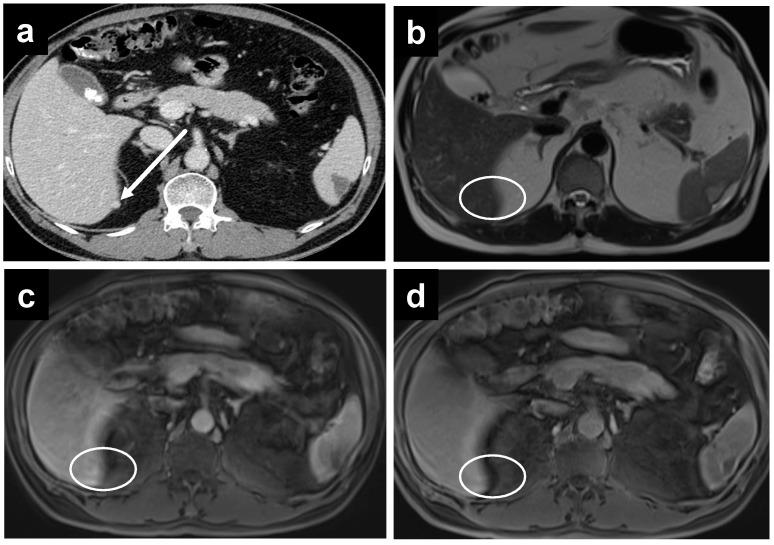
A 63-year-old male with malignant epithelioid mesothelioma. An enhancing perihepatic nodule is well demarcated on (**a**) CT but not visualized on (**b**) T2W, (**c**) portal venous or (**d**) delayed phases on MRI performed on the same day, due to motion artifacts.

**Figure 10:**
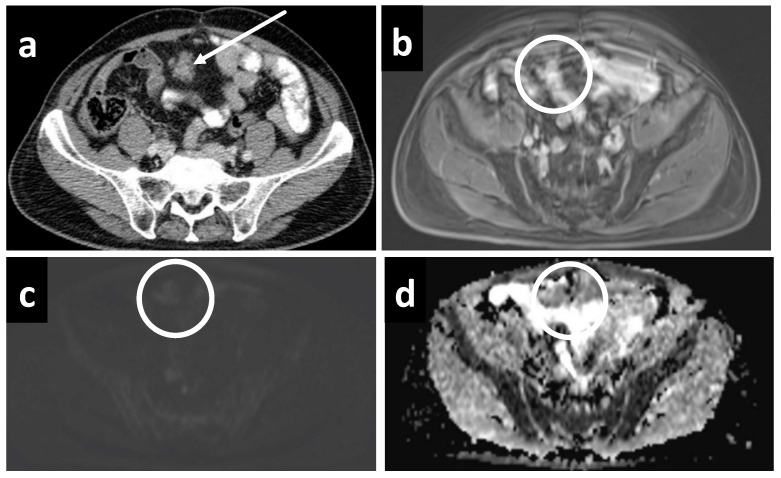
A fairly large soft tissue density peritoneal deposit is well seen on (**a**) CT, as it is surrounded by hypodense fat. Despite its large size, it is obscured on MRI with (**b**) delayed, (**c**) DWI, and (**d**) ADC sequences, primarily due to motion artifacts.

While MRI has seen a considerable increase in use, it does have its drawbacks, including motion artifacts arising from respiration and intestinal peristalsis, logistical concerns, such as high costs and long image acquisition times, as well as a sizable list of contraindications. Moreover, there are certainly scenarios where CT remains the preferred option radiologically. For example, the superior spatial resolution on CT may show enhancing stranding and nodularity better within hypodense fat such, as in the omentum (Figure 8). For the same reason, small enhancing nodules are also occasionally better seen on CT compared to MRI, which in this case is further handicapped by motion artifacts (Figure 9). Sometimes, even a fairly large mass can be difficult to visualize using DW-MRI in the presence of motion artifacts, low tumor cellularity, and when surrounded by relatively hyperintense bowel, despite being well captured on CT (Figure 10).

Taking a step back, it is important to recognize that comparative clinical studies in radiological imaging of PSM is altogether a challenging endeavor. This may be due to heterogeneity in histology and diverse disease manifestation across patients; presentation of PSM can range from nodules and masses of varying size to thin sheets, confluent plaques, as well as omental cake with or without ascites. Certain PSMs also frequently present as mucinous deposits, most commonly those originating from appendiceal and ovarian primaries. Micronodular deposits and thin sheets can be especially hard to visualize on imaging. Furthermore, a lack of clear boundaries between peritoneal regions poses an intrinsic difficulty in radiological PCI assessment. Additionally, comparisons between previous works can also be complicated due to heterogeneities in design and setting, imaging acquisition and processing protocols, characteristics of patient populations, as well as the reporting radiologists’ specialty and experience.

The current study is limited by some constraints, primarily in that it was performed at a single center with a relatively modest sample size. We however note that our findings are largely consistent with various studies performed in other major cancer centers. The strengths of the study include a prospective design; usage of standardized pre-operative protocol; ensuring all patients received both imaging modalities within the appropriate time frame; standardized imaging capture and processing pipeline; as well as image evaluation by three dedicated abdominal radiologists.

Moving forward, it is clear that the ability of radiological imaging modalities to pick up PSM has vastly improved over the years with newer techniques and protocols, and much of the varied results in the literature reflect findings from earlier studies. Continual research and re-evaluation of performance in this arena is therefore definitely warranted as imaging technology develops.

## 5. Conclusions

Imaging for PSM is essential in determining resectability but continues to pose a challenge for the clinician. Our study has demonstrated that the performance of CT and MRI are comparable in the overall detection and evaluation of PSM, although we have additionally sought to offer our experience of specific scenarios where either might be preferred. Given that the current evidence does not strongly suggest the superiority of one imaging modality over another, judicious use based on clinical judgement and resource management may therefore be appropriate at this juncture. Important caveats to note, however, include the underestimation of actual disease burden that is present in both imaging modalities, as well as the somewhat poorer sensitivity of CT for small bowel disease.

## Figures and Tables

**Table 1:** Comparison of tumor detection on imaging against intra-operative findings.

	TP	FN	TN	FP	%Sen	%Spec	%Acc	*p* ^
Total PCI regions								
MRI	110	114	227	17	49.1	93.0	72.0	0.755
CT	107	117	232	12	47.8	95.1	72.4	
PCI region 0—central								
MRI	6	9	21	0	40.0	100.0	75.0	1.000
CT	7	8	21	0	46.7	100.0	77.8	
PCI region 1—right upper								
MRI	13	5	17	1	72.2	94.4	83.3	1.000
CT	12	6	17	1	66.7	94.4	80.6	
PCI region 2—epigastrium								
MRI	6	10	19	1	37.5	95.0	69.4	1.000
CT	7	9	19	1	43.8	95.0	72.2	
PCI region 3—left upper								
MRI	9	5	18	4	64.3	81.8	75.0	0.500
CT	11	3	21	1	78.6	95.5	88.9	
PCI region 4—left flank								
MRI	9	4	19	4	69.2	82.6	77.8	1.000
CT	10	3	21	2	76.9	91.3	86.1	
PCI region 5—left lower								
MRI	12	8	12	4	60.0	75.0	66.7	1.000
CT	12	8	13	3	60.0	81.3	69.4	
PCI region 6—pelvis								
MRI	19	8	9	0	70.4	100.0	77.8	1.000
CT	20	7	8	1	74.1	88.9	77.8	
PCI region 7—right lower								
MRI	11	8	14	3	57.9	82.4	69.4	1.000
CT	12	7	14	3	63.2	82.4	72.2	
PCI region 8—right flank								
MRI	12	8	16	0	60.0	100.0	77.8	0.625
CT	14	6	16	0	70.0	100.0	83.3	
PCI region 9—upper jejunum								
MRI	3	12	21	0	20.0	100.0	66.7	0.250
CT	0	15	21	0	0	100.0	58.3	
PCI region 10—lower jejunum								
MRI	2	13	21	0	13.3	100.0	63.9	1.000
CT	1	14	21	0	6.7	100.0	61.1	
PCI region 11—upper ileum								
MRI	5	11	20	0	31.3	100.0	69.4	0.125
CT	1	15	20	0	6.3	100.0	58.3	
PCI region 12—lower ileum								
MRI	3	13	20	0	18.8	100.0	63.9	0.250
CT	0	16	20	0	0	100.0	55.6	

Abbreviations: TP, true positive; FN, false negative; TN, true negative; FP, false positive; Sen, sensitivity; Spec, specificity; Acc, accuracy; NA, not available; PCI, peritoneal cancer index. ^ McNemar test to compare sensitivity of MRI and CT.

**Table 2:** Agreement between imaging scan and intra-operative PCI score by PCI region.

	Weighted Kappa (95% CI)
PCI region 0—central	
MRI	0.490 (0.220–0.760)
CT	0.586 (0.347–0.825)
PCI region 1—right upper	
MRI	0.612 (0.407–0.817)
CT	0.642 (0.445–0.839)
PCI region 2—epigastrium	
MRI	0.578 (0.333–0.823)
CT	0.567 (0.303–0.831)
PCI region 3—left upper	
MRI	0.622 (0.386–0.857)
CT	0.747 (0.548–0.945)
PCI region 4—left flank	
MRI	0.565 (0.319–0.811)
CT	0.677 (0.448–0.905)
PCI region 5—left lower	
MRI	0.537 (0.315–0.759)
CT	0.501 (0.270–0.731)
PCI region 6—pelvis	
MRI	0.570 (0.359–0.781)
CT	0.578 (0.377–0.779)
PCI region 7—right lower	
MRI	0.439 (0.207–0.671)
CT	0.517 (0.299–0.735)
PCI region 8—right flank	
MRI	0.594 (0.417–0.770)
CT	0.598 (0.421–0.774)
PCI region 9—upper jejunum	
MRI	0.166 (0.040–0.293)
CT	0
PCI region 10—lower jejunum	
MRI	0.220 (−0.038–0.477)
CT	0.182 (−0.115–0.479)
PCI region 11—upper ileum	
MRI	0.277 (0.072–0.482)
CT	0.138 (−0.102–0.378)
PCI region 12—lower ileum	
MRI	0.118 (−0.010–0.246)
CT	0

Abbreviations: CI, confidence interval; PCI, peritoneal cancer index.

## Data Availability

The data presented in this study are available upon reasonable request to the corresponding author.
